# Using short-message-service notification as a method to improve acute flaccid paralysis surveillance in Papua New Guinea

**DOI:** 10.1186/s12889-016-3062-5

**Published:** 2016-05-17

**Authors:** Siddhartha Sankar Datta, Berry Ropa, Gerard Pai Sui, Ramzi Khattar, Ravi Shankar Santhana Gopala Krishnan, Hiromasa Okayasu

**Affiliations:** World Health Organization, Port Moresby, Papua New Guinea; National Surveillance Unit, National Department of Health, Port Moresby, Papua New Guinea; University Health Network, Multi-Organ Transplant Program, Toronto, Canada; World Health Organization, Geneva, Switzerland; University of Toronto, Max Bell Research Centre, 200 Elizabeth St, Room 2-416, Toronto, ON M5G 0A3 Canada

**Keywords:** Disease surveillance, Mobile phone-based surveillance systems, Acute flaccid paralysis, Papua New Guinea

## Abstract

**Background:**

High quality acute flaccid paralysis (AFP) surveillance is required to maintain polio-free status of a country. Papua New Guinea (PNG) is considered as one of the highest risk countries for polio re-importation and circulation in the Western Pacific Region (WPRO) of the World Health Organization due to poor healthcare infrastructure and inadequate performance in AFP surveillance. The Government of PNG, in collaboration with WHO, piloted the introduction of short-message-service (SMS) to sensitize pediatricians and provincial disease control officers on AFP and to receive notification of possible AFP cases to improve surveillance quality in PNG.

**Methods:**

Ninety six health care professionals were registered to receive SMS reminders to report any case of acute flaccid paralysis. Fourteen SMS messages were sent to each participant from September 2012 to November 2013. The number of reported AFP cases were compared before and after the introduction of SMS.

**Results:**

Two hundred fifty three unique responses were received with an overall response rate of 21 %. More than 80 % of responses were reported within 3 days of sending the SMS. The number of reported AFP cases increased from 10 cases per year in 2009–2012 to 25 cases per year during the study period and correlated with provincial participation of the health care professionals.

**Conclusions:**

Combined with improved sensitization of health care professionals on AFP reporting criteria and sample collection, SMS messaging provides an effective means to increase timely reporting and improve the availability of epidemiologic information on polio surveillance in PNG.

## Background

Poliovirus (polio) is a highly contagious pathogen that may cause a life-threatening paralytic disease and polio-induced respiratory insufficiency requiring intubation and mechanical ventilation [1]. Clinical sequaelae include abortive poliomyelitis, aseptic meningitis and paralytic disease, with a mortality rate of 5–15 % primarily due to acute paralytic polio [2–4]. Failure to eradicate the virus may result in the re-emergence and potential outbreak of polio disease in otherwise polio-free areas. The Global Polio Eradication Initiative (GPEI) is a partnership of the World Health Organization (WHO), Rotary International, the US Centers for Disease control and Prevention (CDC), and the United Nations Children’s Fund (UNICEF) and has been successful in reducing the incidence of confirmed cases of polio by 99 % from 1988 to 2013 [5]. As of 2015, wild poliovirus remains endemic in Pakistan, Afghanistan and Nigeria [6]. The success of the GPEI can be attributed to successful vaccination initiatives, improved hygienic practices, training of health care professionals (HCP) to deliver quality immunization services and most importantly, establishing a disease surveillance to detect a possible outbreak. Outbreaks are managed using a live attenuated oral poliovirus vaccine (OPV). To mitigate the risks of re-importation and circulation of poliovirus, a sensitive surveillance system coupled with effective vaccination covering at-risk children are the two most effective methods to promptly contain an outbreak [7]. Environmental sampling from sewage and genetic sequencing are used to distinguish between circulating vaccine-derived polio-virus (cVDPV) and wild poliovirus isolates [8]. Surveillance of acute flaccid paralysis (AFP) cases characterized by acute onset of muscle paralysis in children less than 15 years of age is used as a sensitive indicator of polio infection in the community [9]. Polio-free countries need to maintain a sensitive AFP surveillance and ensure a high OPV immunization coverage through strengthened routine immunization services as part of the polio end game plan of these countries. Therefore, countries are required to report any case of AFP in children aged 15 years and less, with an expected target of 1 case of AFP per 100,000 children under 15 years per year [10]. Furthermore, all cases of AFP must be investigated within 14 days, followed by collection of two stool samples 24 h apart within 14 days after the onset of paralysis. The stool samples are tested in a WHO accredited laboratory to confirm the absence of poliovirus [11]. A 60 day follow-up examination is also required to evaluate the residual paralysis [12]. Papua New Guinea (PNG) is at significant risk of polio re-importation from polio-infected countries and circulation due to poor healthcare infrastructure, inadequate training of healthcare providers (HCP) and insufficient surveillance activities, especially in remote regions [13]. The rate of AFP reporting in PNG has significantly declined in 2000 when PNG was declared polio-free as part of WHO Western Pacific Region (WPRO), failing to reach a target non-polio AFP rate of 1/100,000 children under 15 with poor stool adequacy [14]. The non-polio AFP rate and stool adequacy in PNG between September 2011 and September 2012 was 0.14/100,000 children under 15 and 0.20/100,000 respectively (Data from National AFP Line list). Under-reporting from the provinces in PNG poses a risk to the global polio eradication program because of the difficulty in distinguishing between a failure to report and a true zero-report. In addition to insensitive AFP surveillance, the national routine immunization coverage of Oral Polio Virus 3 (OPV3) in 2011 was only 57 %, coupled with supplementary immunization activities (SIA) coverage of below 80 % in highly populated provinces. With declining AFP surveillance from 2008 to 2012 and suboptimal OPV coverage in routine immunization, the Polio Regional Certification Committee (RCC) of WHO WPRO highlighted PNG as one of the highest-risk countries of polio virus importation from polio-endemic countries [15].

The role of the pediatrician in PNG is critical to the identification, investigation and follow-up of all AFP cases [16]. Newly inducted pediatric medical officers are less sensitized towards reporting cases of AFP, with minimal involvement of Provincial Disease Control Officers (PDCO) in active surveillance and little feedback provided to reporting medical officers on test results, which were identified as critical factors to declining surveillance performance in PNG. Based on increased risk for polio virus importation due to recent economic activities and the success of mobile phone based syndromic surveillance systems in PNG [17], the Polio National Certification Committee (NCC) recommended that the National Department of Health (NDoH) should pilot the use of mobile-phone based alert systems (SMS Alert) to improve the detection and reporting of AFP cases, to raise awareness of AFP surveillance among pediatricians and the PDCOs and to increase timely reporting with support from WHO-PNG. In this report, we demonstrate the results of the implementation of SMS messages to sensitize the pediatrician and the provincial disease control officers in reporting of AFP cases, which also indicates a cost-effective means to increase surveillance reporting of AFP and other communicable disease in the NSS in PNG.

## Methods

### Participants

The SMS Alert pilot introduction took place from September 2012 to November 2013. From September 2012 to February 2013, the SMS Alert was sent to 44 pediatricians to seek information whether they had seen any case of an AFP in their clinic or community. Following the initial success of the project, the NDoH also enrolled PDCO, infection control officers (ICO), field epidemiology training program officers (FETP) and other provincial and district officers in March 2013. As a result, a total of 96 healthcare professionals and officers were registered to receive bi-monthly SMS reminders to report any AFP case, including 44 pediatricians, 14 FETPs, 17 provincial hospital ICOs, 13 PDCOs, 4 NDoH officers and 4 other healthcare professionals. All 20 provinces of PNG except for East Sepik had at least one registered official. Of the provinces that registered in SMS Alert, 12 provinces had either a pediatrician or a PDCO registered, while 7 other provinces had representation of both PDCO and pediatricians.

### SMS message

SMS services were transmitted using services of Digicel Mobile Service provider in Papua New Guinea, which had an initial cost of 45 USD for the modem and 50 USD per month for the cost of SMS. The Frontline SMS software was used to manage and generate automated SMS messages. Frontline SMS software can be utilized without the need for an internet connection and uses a GSM modem/internet dongle with a local phone number to send and receive SMS messages. In each SMS message, participants were asked if they had reviewed a case of AFP over the last two weeks and were voluntarily asked to give their response, 1 for yes and 0 for no. The SMS message also allowed communication on the follow-up action after identification of the cases, which also provided a platform for repeated sensitization/orientation of the professionals.

### Analysis

To assess the impact of SMS Alert on the AFP surveillance, the following indicators were monitored and analysed including, a) participation rate, b) SMS response time, c) changes in number of reported AFP cases and stool adequacy. Participation rates were defined as the average percentage of responses from PDCO or pediatrician to the 14 SMS messages sent during the data collection period. SMS response times were determined by measuring the time of response from the time the SMS messages were sent. Notification of any AFP cases received by SMS was forwarded to the National Surveillance System (NSS) unit for follow-up with provinces and provincial hospitals. All cases that were notified by SMS were also reported to the NSS. The number of reported AFP cases between 10/9/2008 and 9/9/2013 in the NSS was obtained from the National AFP line-list. To evaluate the effectiveness of SMS Alert, analysis was controlled temporally in a before and after study comparing data collected prior to the introduction of SMS technology and following the introduction of the pilot study. Maps were generated using the Administrative Boundaries: World Health Organization Base Map: ESRI and produced by WHO headquarters, Geneva. Stool adequacy was defined as cases that had two stool samples collected 24 h apart within 14 days after the onset of paralysis.

## Results

### Participation in SMS alert

**Fig. 1:**
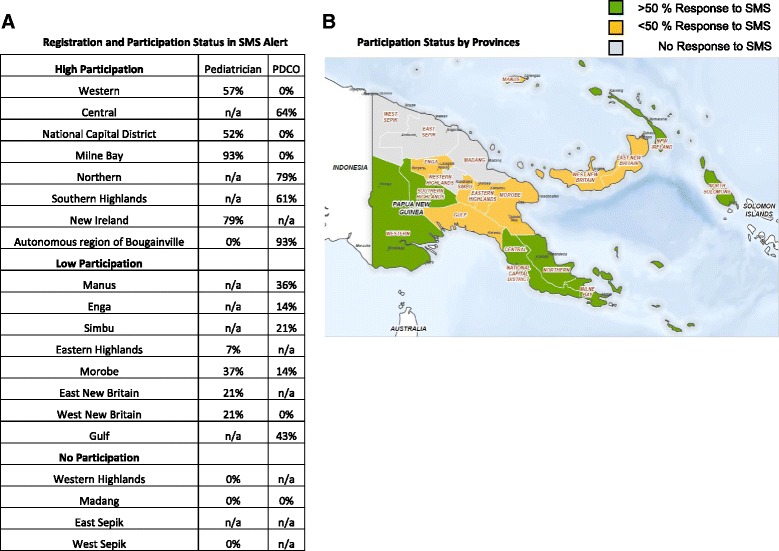
Participation of informants in provinces of Papua New Guinea in SMS Alert. Pediatrician or PDCO participation was ascertained based on response rate. High Participation was defined as responses of greater than 50 % of SMSs, while low participation was defined as responses of less than 50% of SMSs (**a**). The distribution of participation is shown in a provincial map of Papua New Guinea (**b**)

Fourteen SMS messages were sent to 96 registrants with 117 unique phone numbers on a bi-monthly basis. While all 96 registrants received SMS messages, responses were expected from 88 registrants. A total of 432 responses with 253 unique responses were received with an overall response rate of 21 % (253 unique responses/1232 total SMS sent). Thirty-seven percent of the responses consisted of duplicate messages with multiple responses from the same individual using multiple phones and other communication. In provinces that had both PDCO and pediatricians registered in the SMS Alert system, responses were received from either the PDCO or the pediatrician with the exception of Morobe province, which had responses sent from both PDCO and pediatricians. Figure 1a-b demonstrates the distribution of provincial participation in SMS Alert across PNG.

### Response time

Of the SMS that were received from the participants, more than 80 % of responses were reported within 3 days of sending the SMS. While SMS were sent from the National level in a timely manner, more than 50 % of pediatricians, PDCO and ICO received SMS messages, but never replied to any of them. More than 60 % of responses were not in the requested format of 0 for no cases of AFP and 1 for cases of AFP. The responses that did not comply with the requested format included case summaries or sentence responses such as, “NIL cases found”.

### Changes in number of reported AFP cases and stool adequacy

**Fig. 2:**
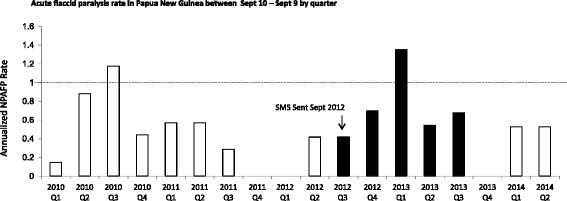
SMS notifications increase AFP reporting in Papua New Guinea. The number of AFP cases reported in the AFP line list 2010–2014 are shown by yearly quarters. The black dotted line indicates required surveillance performance indicator targets

Despite poor compliance with reporting format and lack of responses from some of the participants, AFP reporting in PNG increased when SMS notification were sent. A 6-fold increase in reporting of AFP cases was observed from September 2011 to September 2012 and September 2012–September 2013. Prior to SMS notifications, the average number of AFP cases reported was 10 +/− 3.3 (Non-Polio AFP rate = 0.37 +/− 0.12) from September 2009 to September 2012, which increased to 25 AFP cases reported (Non-Polio AFP rate = 0.85) following SMS notifications. Quarterly analysis further revealed that as the program was expanded to participants beyond the pediatricians in March 2013, there was more reporting of cases in the NSS, reaching its peak in the first quarter of 2013 (Fig. 2). However, when the initiative culminated in November 2013, reporting of AFP cases dramatically dropped to zero in the fourth quarter of 2013. Moreover, comparing the pattern of reporting both pre- and post- evaluation period of SMS Alert, there was an observable trend of improved reporting of AFP cases from the provinces with better representation and participation in this SMS initiative. Interestingly, there was also an increase in reporting of AFP cases even from the provinces without any response, suggesting that receiving regular SMS notifications alone may have acted as a sensitization tool, thereby instigating reporting.

The increase in reporting to using SMS Alert did not result in reducing the time period for investigation, nor the time for collection of 2 stools from the date of onset of paralysis. The stool adequacy also declined during the time period of the initiative.

## Discussion

This study demonstrated that sensitization through repeated reminders can improve reporting of AFP cases in PNG and concomitantly encourages HCP to promptly report all cases of AFP in real-time to the NSS. The results further highlight that responding to SMS Alert correlated with improved AFP surveillance in the province. Interestingly, AFP reporting improved in provinces where responses were not sent back. This may be due to the sensitization effect of SMS, as similar effects have been observed in SMS use for AFP surveillance other countries [18] as well as monitoring of dengue fever in PNG [17]. Several studies have demonstrated that sensitization by polio vaccination campaigns and polio outbreaks [4, 19–21] in adjacent countries can increase reporting of AFP. Ndiaye et al. demonstrated that increased community awareness of the symptoms of AFP can enhance AFP surveillance and reduces resistance to polio vaccination [18]. This research also demonstrates a potential solution to the important issue of declining AFP surveillance post-eradication in countries that are at risk for polio importation.

PNG has unique challenges in its disease surveillance system with 87 % of its population inhabiting rural communities and only 3 % of its roads paved [17]. Inadequate healthcare, geographic isolation, poor communication network and infrequent transportation make disease surveillance in PNG particularly difficult. Healthcare accessibility and the availability of timely epidemiological information remains a challenge in PNG, where significant mortality still occurs from communicable diseases such as, malaria, tuberculosis, diarrheal diseases and acute respiratory infections [22]. Nonetheless, in these circumstances, disease surveillance remains critical to improve preparedness and allows rapid assessment of the extent of disease outbreaks including determining the dynamics of outbreak response.

PNG has been declared polio free since 2000, however it remains at high risk for polio virus importation, as surveillance targets and vaccination coverage target do not meet the World Health Organization standards. Furthermore, under-reporting from the provinces in PNG signals inadequate disease surveillance, making it difficult to distinguish between a failure to report and a true zero-report. Under the standard system of AFP reporting, provincial hospitals act as AFP sentinel sites with a small number of cases being reported to the NSS from health centres, which are often referred to provincial hospitals for effective case management and investigation. Reporting to the NSS is done by phone or fax. Stool sample collections are processed by Provincial Disease Control Officers [17]. Given the limited infrastructure and scattered populations, SMS Alert may be a useful tool to improve AFP disease surveillance and mitigate the risks of re-importation. Using SMS Alert, reporting is still encouraged to the NSS, but there is opportunity for missed cases to be reported through SMS with the added benefit of increasing sensitization to the symptoms of AFP.

While the findings of this pilot are encouraging, suggesting an improvement in AFP surveillance, it is possible that this improvement is due to the improving the overall quality of national program (such as other sensitizing efforts) or the observed improvement is temporary. A longitudinal study over several years is required to conclusively prove whether the SMS Alert has long-term benefit.

Expansion of the SMS Alert initiative to cover other medical officers and HCP in the country is recommended to improve AFP surveillance and assess the long-term benefit of the SMS Alert. While responses to SMS were sent in a timely manner, it was observed that 49 % of registrants expected to send text messages failed to respond to any SMS messages. Further analysis and interviews with the key participants will be required to identify and address the reasons for failing to report through SMS. In addition, it may be helpful to follow up individually with those who did not respond (e.g., by phone) to obtain responses.

A system to update the contact information of the participants or to add newly deployed HCPs may improve both participation and response rates. Moreover, cases that are notified by SMS should be followed up and investigated to improve timely investigation and timely stool sample collection. Improvements in the existing SMS system can be addressed to enhance the quality of data reporting by SMS. These include making suitable provisions to accept only one answer from one phone number to reduce the number of duplicate responses and forcing answers into a 0 or 1 format only, or requesting informants to re-send the response if the response is not in the required 0 or 1 format. While other responses were accepted and included in the analysis for this report, the 0 or 1 format will aid with automation of reports if SMS Alert were to be expanded in PNG.

Sensitive acute flaccid paralysis surveillance is used to monitor suspected cases polio disease in a community. Unexpected increases in AFP cases can be predictive of an impending polio outbreak [23], however, laboratory testing of adequately collected stool from suspected cases is required to confirm the presence of the virus. In this study, markers of system performance including the time period of investigation of the reported AFP cases and stool adequacy failed to reach the surveillance targets possibly due to the increased burden on follow-up and coordination upon an already poor healthcare infrastructure. The time period in investigation of all the reported cases was observed to have widened following the introduction of SMS Alert and stool adequacy rate fell, underscoring the necessity of increased investment into the healthcare system and training of HCP including better coordination role of the NSS if it is to manage the increase in number of cases as generated by the SMS Alert system.

A surveillance review at Wewak and Maprik Provincial Hospitals in PNG in 2012 revealed that some of the AFP cases although admitted in the hospitals were not reported to NSS due to lack of pediatricians' awareness of AFP reporting criteria, suggesting that SMS alone does not necessarily solve the surveillance issue and will only be useful when the SMS Alert system is combined with training and re-sensitization of HCP on AFP and other vaccine preventable disease surveillance. Combined with improved training of pediatricians and PDCOs on AFP reporting criteria, sample collection and addressing the current immunity gaps in high risk regions, SMS Alert provides an effective means to enhance surveillance in remote regions of PNG.

## Conclusions

Collectively, the results from SMS alert initiative highlight the importance of training and sensitization on quality AFP surveillance in a country; more so in PNG where the country is identified at high risk to any polio virus importation. Coupled with increased investment in training, SMS Alert is considered as a highly cost-effective strategy to enhance AFP surveillance in remote areas of PNG and may be effective in improving surveillance in resource constrained polio-endemic regions. Improved AFP surveillance will provide the timely epidemiologic information to direct supplementary immunization activities and mitigate the risks of viral spread and importation. If proven effective, mobile-phone strategies such as, SMS Alert could be implemented to increase the surveillance of other communicable diseases, preventing disease spread and reduce morbidity and mortality of population in remote communities.

### Ethics

Ethical approval and consent to send SMS health care professionals was approved by the National Department of Health of Papua New Guinea. Informed consent to use and publish the results was received from health care professionals participating in this pilot study. Informed consent was received from health care professionals participating in the study.

### Consent to publish

The results have been approved for publication by the National Department of Health in Papua New Guinea by Dr. Sibauk Vivaldo Bieb, Executive Manager of Public Health of the Government of Papua New Guinea.

## Abbreviations

AFP: acute flaccid paralysis
FETP: field epidemiology training program officers
GPEI: global polio eradication initiative
HCP: healthcare providers
ICO: infection control officers
NDoH: National Department of Health
NSS: National Surveillance System
OPV3: Oral Polio Virus 3
PDCO: provincial disease control officers
PNG: Papua New Guinea
SIA: supplementary immunization activities
SMS: short-message-service
UNICEF: the United Nations Children’s Fund
WHO: World Health Organization
WPRO: Western Pacific Region
